# Bilateral Polycystic Kidney Disease and Inferior Vena Cava (IVC) Thrombosis: A Cadaveric Study

**DOI:** 10.7759/cureus.80765

**Published:** 2025-03-18

**Authors:** Gongchao Yang, Morgan E Schock, Peyton W Wall, Yuefeng Lu, Dongmei Cui

**Affiliations:** 1 Department of Advanced Biomedical Education, University of Mississippi Medical Center, Jackson, USA; 2 School of Medicine, University of Mississippi Medical Center, Jackson, USA

**Keywords:** autosomal dominant polycystic kidney and liver disease, cadaver case report, clinical implication, inferior vena cava filter (ivcf), polycystic kidney and liver disease

## Abstract

Autosomal dominant polycystic kidney disease (ADPKD) is the most common genetic kidney disease and one of the most common causes of end-stage kidney disease. The occurrence of bilateral polycystic kidney disease with liver cysts is uncommon. A case of bilateral polycystic kidney disease, found to have liver cysts and inferior vena cava thrombosis in a cadaveric study, is presented in this study. Dissection was performed on a 90-year-old Caucasian male cadaver. Both kidneys were enlarged and contained multiple cysts with normal renal tissue interposed. When comparing the two kidneys, the cysts on the left were notably larger. There was a partial absence of renal capsules. Additionally, a polycystic liver and an inferior vena cava filter were found during the dissection. The patient's history of venous thrombosis is indicated by the previously inserted inferior vena cava filter.

## Introduction

Autosomal dominant polycystic kidney disease (ADPKD) is the most common genetic kidney disease that is mostly present in adults and one of the most common causes of end-stage kidney disease in human, affecting up to 12 million individuals worldwide [1]. ADPKD is a multisystem disorder that is characterized by gradually growing renal cysts that begin developing before birth and can originate from all areas of the kidneys. However, cysts usually form in the distal regions of the nephron and the collecting duct [2,3]. The disease is both progressive and incurable, and it has a significant risk of cardiovascular complications. The renal phenotype in patients with ADPKD ranges from patients in old age without renal failure to rare cases of enlarged kidneys that are detected in utero [4]. Cysts in patients with ADPKD are fluid-filled focal outgrowths from the renal epithelium. Numerous cellular changes have been observed in cyst-lining cells. The changes include alterations in the differential distribution of phospholipid-protein complexes and cytoskeletal components between the apical area and basal area, the protein-mediated signaling that regulates the orientation of cells in a layer of epithelial tissue, increase in the production of the extracellular matrix, and cellular metabolism. These changes can affect essential cellular functions such as fluid transport, proliferation, apoptosis, and cell adhesion and differentiation [1]. A majority of patients develop arterial hypertension which contributes to the increased cardiovascular morbidity and mortality in the early course of ADPKD when renal function is still normal [1,3]. The diagnosis of ADPKD can be made using abdominal imaging such as ultrasound, computed tomography (CT), or magnetic resonance angiography (MRA). These modalities are used to determine total kidney volume and assess the progression of disease in the clinic [5]. Polycystic liver disease is the most common extrarenal expression [6]. Simple and solitary cysts are typically observed, while complex hepatic cysts are less common. The normal hepatic parenchyma and liver function are usually preserved. Rare complications may be related to enlarged liver cysts [7]. Liver cysts often remain asymptomatic; however, the increasing volume of cysts in some individuals may result in the massive enlargement of the liver, causing abdominal pain, back pain, early satiety, dyspnea, malnutrition, and a significant impairment in quality of life [8-12].

The case we presented in the study of cadavers is characterized by bilateral polycystic kidney disease, liver cysts, and inferior vena cava (IVC) thrombosis.

## Case presentation

We present a 90-year-old Caucasian male cadaver with bilateral kidney enlargement. A brief medical history of this male donor included hypertension, cardiac bypass, hernia, interstitial pulmonary disease, Parkinson's disease, and hypercholesterolemia. The cause of death was cardiac arrest and interstitial lung disease. 

**Figure 1:**
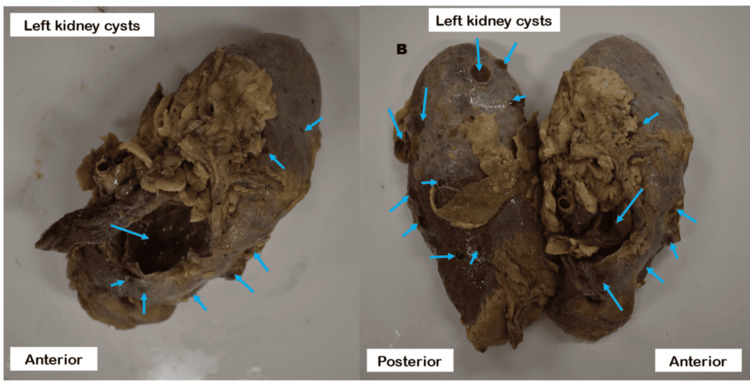
Left kidney cysts Cysts appear on the anterior and posterior surfaces of the left kidney.

The parietal peritoneum along the lateral side of the ascending colon was cut open and removed. The ascending colon was reflected medially. Blunt dissection was used to remove the pararenal fat, renal fascia, and perirenal fat to expose the right kidney. Similarly, these steps were repeated for the side of the descending colon to expose the left kidney. The bilateral renal capsules were removed. The kidneys were examined by means of visual inspection, dissection, coronal section, and photography. When comparing the two kidneys, the cysts on the left were notably larger. The right kidney is 12.24 cm long and 6.59 cm wide. The left kidney is 16.32 cm long and 7.68 cm wide. Both the left and right kidneys contain multiple cysts and interposed normal renal tissue. A dozen cysts of various sizes are visible along the coronal plane of the left kidney, and the largest cyst measures 2.72 cm × 3.26 cm. A cyst measuring 5.44 cm × 3.26 cm is located on the anterior surface of the left kidney near the inferior pole. The posterior surface of the left kidney has multiple cysts that are less than 2 cm in diameter, as shown in Figure 1.

**Figure 2:**
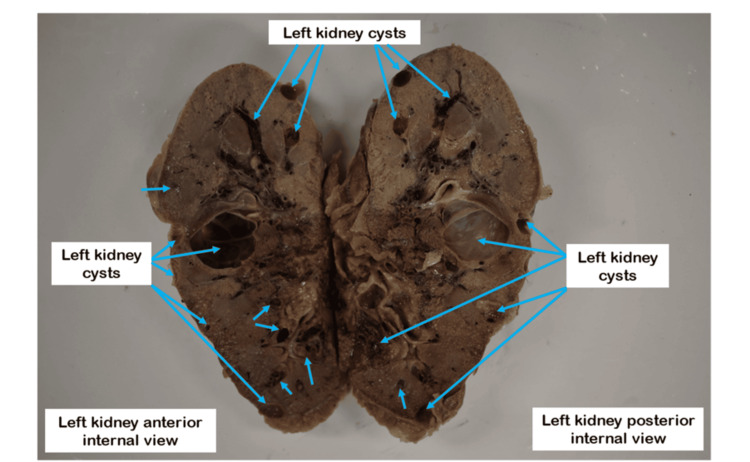
An inside view of the left kidney on the coronal plane Blue arrows indicate cysts that appear on the coronal plane of the left kidney.

There was a partial absence of renal capsules on the posterior inferior surface of the left kidney. An amber-colored secretion is visible in the cysts of the left kidney. The coronal plane of the left kidney has bloodstains that indicate bleeding into some cysts and the renal sinus (Figure 2).

**Figure 3:**
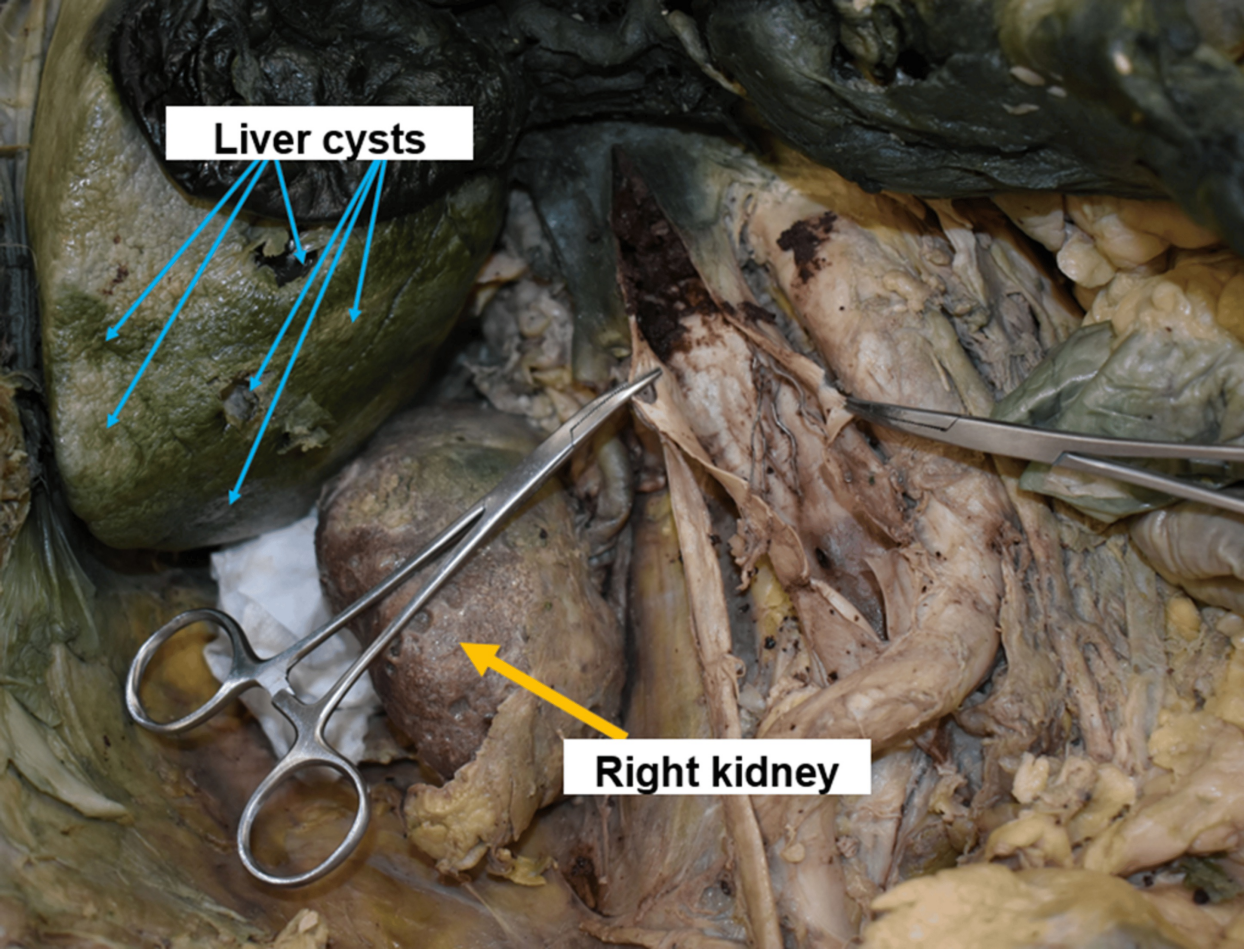
Kidney and liver cysts Blue and yellow arrows indicate the presence of six liver cysts and right kidney cysts.

The anterior surface of the right kidney is lined with multiple small cysts (Figure 3).

**Figure 4:**
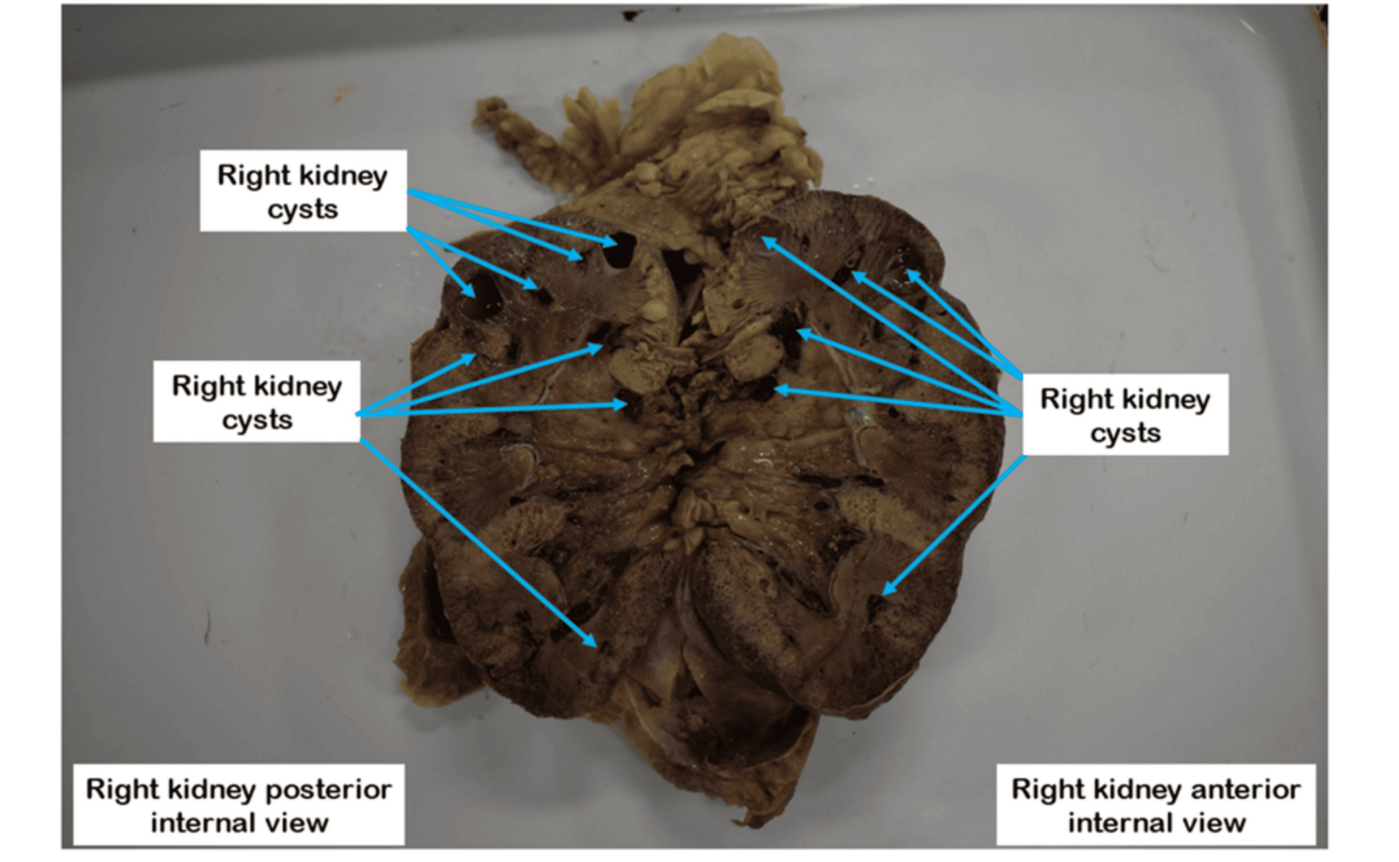
Right kidney cysts on the coronal plane Blue arrows indicate cysts that appear on the coronal plane of the right kidney.

Multiple small cysts are present in the coronal section of the right kidney, some of which have clear cyst membranes and fluid while others are bloodstained (Figure 4).

**Figure 5:**
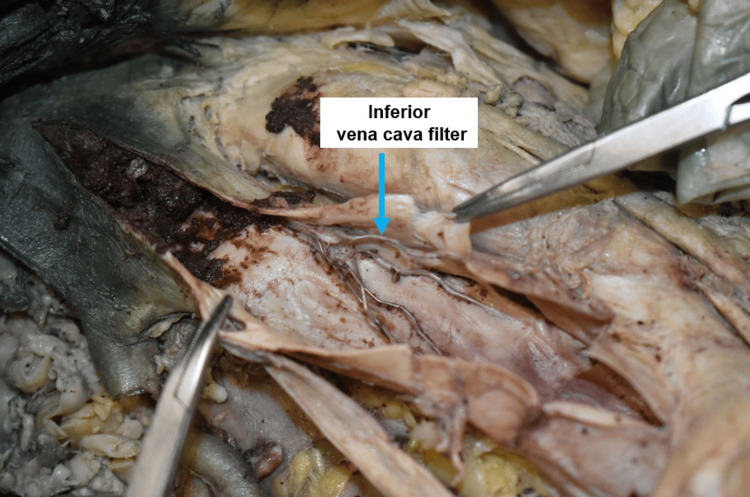
Inferior vena cava filter A filter is located within the inferior vena cava and contains a blood clot.

Furthermore, six distinct cysts were found on the liver's visceral surface. The largest cyst ruptured and measured 3.2 cm in diameter. Dark blue secretions and a blood clot can be seen in the ruptured cyst. Other cysts were less than 3 cm in diameter. There were depression and softness in the locations of the other cysts. The liver's visceral surface showed nodular bumps (Figure 3). The pathological organs were compared to the normal kidneys and livers. A filter was discovered through the palpation of the IVC. Dissecting the IVC revealed a Greenfield filter, a conical stainless-steel wire filter that measures 4.6 cm. The IVC filter shifted to the left and collapsed because of compression. The superior IVC to the IVC filter was filled with blood clots. Records regarding filter placement were unavailable (Figure 5).

The liver's visceral surface showed nodular bumps. Six distinct cysts were identifiable on the visceral surface. One cyst ruptured, and the locations of the other cysts were depressed and soft. Evidence of a blood clot was found inside the ruptured cyst (Figure 3).

## Discussion

The findings of this case presentation are similar to those reported by Maeda et al., in an unusual case of IVC thrombosis caused by enlarged cysts in ADPKD [13]. IVC filters have been used to manage patients with pulmonary embolism and deep venous thrombosis [14]. Thrombosis in the IVC due to intra-cystic hemorrhage into a hepatic local cyst with ADPKD was reported by Iguchi et al. [15]. The presence of an IVC filter suggests that the donor had venous thrombosis in his history. In 1984, the Greenfield filter was introduced to solve the issues of filter migration and caval thrombosis. When fully expanded, the filter measures 4.6 cm in length and 3 cm in diameter at its base. The filter can be effectively secured to the caval wall (Figure 5) using the conical stainless-steel wire prongs [16]. This cadaver's findings suggest that the stainless-steel wire in the Greenfield filter is not strong enough to resist the pressure from the surrounding structures. The right kidney is close to the IVC, and the filter has shifted to the left. The collapse of the IVC filter might indicate compression from the enlargement of the right kidney.

The cadaver presented with bilateral kidney cysts and liver cysts, suggesting that the donor had polycystic kidney disease with extrarenal manifestations of polycystic liver disease. In a gross anatomy lab setting, polycystic kidneys might be encountered in cadavers. Secretion and bleeding in the cysts and renal sinus can lead to hematuria and proteinuria. Enlargement of the kidney and liver, loss of the renal capsule, and bleeding could lead to abdominal pain, back pain, early satiety, and dyspnea. It would be beneficial for faculty members to be able to explain the condition to the students, especially with the increasing implementation of system-based health science courses. A typical case of polycystic kidney disease provides a good learning opportunity for the students to not only visualize gross abnormalities but also relate this multi-organ-system condition to many disciplines, including genetics, histology, biochemistry, physiology, and pathology. Students can compare diseased and non-diseased kidneys, validate ultrasound and radiographic imaging, and create a foundation for future clinical study of polycystic kidney diseases. This integration may enhance their understanding of the connection between hypertension and polycystic kidney disease, changes in pathophysiology, and the effects of medical procedures on patients. Due to the progressive nature of ADPKD, students can appreciate how periodic monitoring may be of benefit to patients and why kidney transplantation is the best kidney gold standard therapy for patients with ADPKD who have progressed to end-stage renal disease (ESRD) [17-19].

## Conclusions

ADPKD may be encountered by medical students during their gross anatomy lab experience. In a typical educational setting, students with the opportunity to see this condition may not fully appreciate the breadth of the pathology before them. Emphasis from a faculty member can focus students on the genetic causality, progressive nature, and relationships of multiple organ systems involved with the disease. The experience gained from integrating systems-based associations with cadaver dissection will help students gain a better understanding of this genetic disease, and it will provide a basic science foundation for future clinical studies.
